# Role of Front-of-Package Gluten-Free Product Labeling in a Pair-Matched Study in Women with and without Celiac Disease on a Gluten-Free Diet

**DOI:** 10.3390/nu11020398

**Published:** 2019-02-14

**Authors:** Wioleta Zysk, Dominika Głąbska, Dominika Guzek

**Affiliations:** 1 Department of Organization and Consumption Economics, Faculty of Human Nutrition and Consumer Sciences, Warsaw University of Life Sciences (SGGW–WULS), 159C Nowoursynowska Street, 02–776 Warsaw, Poland; wioleta_zysk@sggw.pl; 2 Department of Dietetics, Faculty of Human Nutrition and Consumer Sciences, Warsaw University of Life Sciences (SGGW–WULS), 159C Nowoursynowska Street, 02–776 Warsaw, Poland; dominika_glabska@sggw.pl

**Keywords:** front-of-package labeling, celiac, gluten-free, gluten-free bread, choice experiment (CE)

## Abstract

Gluten-free (GF) product labeling is one of the most important determinants of food product choices by patients with celiac disease, due to the need for following a GF diet. The aim of this study was to assess the role of front-of-package GF product labeling in pair-matched celiac and non-celiac women on a GF diet in a choice experiment (CE). In subgroups of celiac (*n* = 77) and non-celiac pair-matched respondents on a GF diet, but with no gluten-related diseases diagnosed (*n* = 77), the influence of front-of package labeling of GF bread on the choice of products was assessed. The labeling assessed in a CE included for all the products crossed grain logotype and additional logotypes of European Union (EU) organic production, “dairy-free” product, wheat starch-free product, quality and vegan product, as well as additional “gluten-free” written information. It was stated that the frequency of selection of products with “gluten-free” written information did not differ between subgroups of celiac and non-celiac respondents, as well as in subgroups stratified by age, body mass index (BMI), place of residence, and economic status. The frequency of selection of products with “vegan” logotype was higher for non-celiac respondents than for celiac ones (*p* = 0.0011). The frequency of selection of a product with additional logotypes was influenced by BMI and place of residence, but not by age and economic status of assessed women.

## 1. Introduction

A gluten-free (GF) diet is a therapeutic necessity in patients diagnosed with celiac disease and other gluten-related disorders [1]. Currently, the GF diet is popular not only among patients with gluten-related diseases, but also among individuals who believe it has a beneficial effect to alleviate their other disease despite it not being specifically demonstrated [2]. The GF diet is popular among athletes, who believe it plays a beneficial role in the fitness regimen [3]. However, the GF diet is associated with possible adverse effects in individuals without proven gluten-related diseases [4], because of the risk of nutritional deficits without additional benefits [5]. This is especially important because GF products are, in general, neither fortified nor enriched [6]; therefore, a GF diet based on the same food groups as the patient’s earlier diet [7] may have lower nutritional value.

The considerable interest in GF diets [4] led to sales growth of GF products, with a resultant doubling of their retail sales in the United States since 2011 [8]. The *Codex Alimentarius* of the Food and Agriculture Organization of the United Nations (FAO) and the World Health Organization (WHO) of 1979 (most recent amendment in 2015) [9] introduced a unified definition of GF products as those that consist of, or are made from, ingredients derived from wheat, rye, barley, or oats but are processed to remove gluten or other ingredients, with the gluten level not exceeding 20 mg/kg. A unified, standard definition and labeling were necessary to facilitate safer and informed food product choices by consumers [10]. Simultaneously, however, consumers without gluten-related diseases choose GF products because of perceived beneficial effects that, in fact, cannot be obtained [11]. The popularity of GF products results in their being perceived as health-promoting [12] by one in every three Canadians [13] and every four Americans [14].

According to the European Food Information Council (EUFIC) [15], the general determinants of choice of food products include biological (e.g., hunger), economic (e.g., income), physical (e.g., accessibility), social (e.g., family), and psychological (e.g., mood) factors, as well as attitudes, beliefs, and knowledge. Taking this into account, the determinants of choice for GF products may differ from that for other products, not only because of the attitudes, beliefs, and knowledge of consumers, but also because of the limited availability and variety of GF products, as well as high price [16] (even as much as 10 times higher than for non-GF products) [17]. However, issues with the labeling of GF products pose one of the major barriers in adherence to a GF diet [18]. The risk of gluten contamination, despite being uncommon [19], may pose an important burden for individuals on a GF diet [20]—especially those with lower affordability for certified high-quality products [21]. Thus, concerns associated with the purchase of GF products may influence the general quality of life [22]. Therefore, it is important to analyze additional factors that may influence the choice of GF products. This study was conducted to assess the role of front-of-package GF product labeling in pair-matched women with and without celiac disease on a GF diet in a choice experiment (CE).

## 2. Materials and Methods 

### 2.1. Ethics Approval Statement

The study was conducted according to the guidelines of the Declaration of Helsinki. The study protocol was approved by the Ethics Committee of the Faculty of Human Nutrition and Consumer Sciences of the Warsaw University of Life Sciences (No. 20/2017; 19.06.2017). All the participants provided their informed consent to participate in the experiment.

### 2.2. Study Participants

This study was conducted in a pair-matched cohort of women with and without celiac disease recruited during free GF-diet workshops conducted in all regions of Poland. All participants of the workshops were invited to participate. Because of the pair-matched study design, the recruitment procedure was conducted in two stages. During the first stage, participants were assessed by inclusion and exclusion criteria. In the second stage, participants who qualified for study inclusion were subjected to a random pair-matching procedure; only those who were pair-matched then participated in the study and in the procedure of choice experiment (CE). 

In the first stage of the recruitment procedure, the following inclusion and exclusion criteria were applied:

(1) Inclusion criteria for the celiac GF-diet group:
  - Caucasian;
  - Women;
  - Celiac disease diagnosed and biopsy-confirmed by a physician;
  - GF diet followed for at least half of a year recommended by physician/dietitian due to diagnosed celiac disease;
  - Regular purchase of GF products declared.
(2) Exclusion criteria for the celiac GF-diet group:
  - Lack of consent agreement for participation;
  - Any data missing in the completed questionnaire (forefending against pair-matching procedure).
(3) Inclusion criteria for the non-celiac GF-diet group:
  - Caucasian;
  - Women;
  - GF diet followed for at least half of a year, based on own decision, from reasons other than celiac disease or other gluten-related disease (i.e., non-celiac gluten sensitivity, gluten ataxia, wheat allergy, dermatitis herpetiformis);
  - Regular purchase of GF products declared.
(4) Exclusion criteria for the non-celiac GF-diet group:
  - Celiac disease diagnosed;
  - Any other gluten-related disease diagnosed;
  - Lack of consent agreement for participation;
  - Any data missing in the completed questionnaire (forefending against pair-matching procedure).

Furthermore, in the second stage of the recruitment procedure, the following pair-matching criteria were applied (random purposive sampling):

- Age: for pair-matching, the difference between a celiac GF-diet participant and non-celiac GF-diet participant was set at ≤5 years;
- Body mass index (BMI): for pair-matching, the difference between a celiac GF-diet participant and non-celiac GF-diet participant was set at ≤1.5 kg/m^2^, but only within the same category of malnutrition/proper body mass/excessive body mass;
- Place of residence: for pair-matching, the difference between a celiac GF-diet participant and non-celiac GF-diet participant was set at ≤1 category (for categories of village/town <20,000 residents/city 20,000–100,000 residents/ city >100,000 residents);
- Economic status: for pair-matching, the difference between a celiac GF-diet participant and non-celiac GF-diet participant was set at ≤1 category (for categories of very bad/bad/average/good/very good).

In cases where the celiac GF-diet participant had more than one potential non-celiac GF-diet participant who fulfilled the criteria of pair-matching, the pair-matched participant was randomly chosen.

Six hundred potential respondents were assessed by the inclusion and exclusion criteria, as well as the pair-matching criteria; a specific questionnaire was applied to assess the necessary characteristics. Following the screening procedure, 234 respondents were stated to fulfill the criteria and following the pair-matching, 156 respondents were qualified (78 celiac GF-diet respondents, 78 individually pair-matched non-celiac GF-diet respondents). Due to the fact that, after the pair-matching procedure, only one respondent in each group declared bad/very bad economic status, it was decided to exclude them from the further analysis to reduce the bias, and the final analysis was conducted for a group of 154 respondents (77 celiac GF-diet respondents, 77 individually pair-matched non-celiac GF-diet respondents). Characteristics of age and BMI of study participants stratified by study groups are presented in Table 1; because of pair-matching, there were no between-group differences in age and BMI.

The stratification of the pair-matched celiac GF-diet respondents and non-celiac GF-diet respondents is presented in Table 2; because of pair-matching, there were no between-group differences in stratified age, BMI, place of residence, and economic status.

### 2.3. The Procedure of the Choice Experiment (CE)

The CE is commonly applied to assess food product choice, as it is a relatively simple method that allows obtaining the reliable data associated with simulation of the real market choices [23,24]. As a result, it allows predicting the consumer decisions [25]. Some CEs were also conducted specifically for the labeling of food products in order to specify how the labeling influences the purchase decisions [26]. Taking this into account, the CE method was chosen in order to obtain the most reliable information about the choice of products, depending on the applied front-of-package labeling.

The CE was based on a simple purchase decision when shopping for GF bread. Bread was chosen as a basic product, which is generally commonly applied, but challenging for individuals following a GF diet [27]. Each participant received an identical set of 12 photographs of identical product but with different front-of-package labeling, together with a simple question about which one she would choose (single-choice question) and the reason for her choice (open-ended question transferred into a multiple-choice stratification of answers). The photographs were shown in a random order and presented the same bread packed in transparent packaging, but with different logotypes. For each presented product, the logotypes included the Crossed Grain (CG) logotype, as in accordance with the Crossed Grain Trademark (CGT), applied in Poland under the European licensing scheme of the Association of European Celiac Societies (AOECS) [28]. However, additional logotypes were included, and, for some products, the CG mark was accompanied by additional “gluten-free” written information. The applied attributes were chosen on the basis of the interviews with the celiac disease patients and of expert opinions, followed by the focus group. The attributes were afterward verified in order to be understandable, as well as presented in a clear and concise manner. The scheme of the CE is specified in Table 3, along with detailed product characteristics in random order.

No additional information about ingredients, nutritional value, or price was indicated on the packaging. 

### 2.4. Statistical Analysis

The statistical analysis included the Shapiro–Wilk test to assess the normality of distribution, Mann–Whitney *U* test (for nonparametric distributions) to compare age and BMI in subgroups. The chi-square test was used to compare the stratification of participants in the subgroups, and additional multivariate logistic regression analysis was conducted for all the variables, while the following variables were analyzed: age, BMI, place of residence, and economic status.

All statistical analyses were conducted using Statistica, version 8.0 (Statsoft Inc., Tulsa, OK, USA) and Statgraphics Plus for Windows 4.0 (Statgraphics Technologies Inc., The Plains, VA, USA); *p* ≤ 0.05 was accepted to indicate the level of significance. 

## 3. Results

### 3.1. Influence of Front-of-Package “Gluten-Free” Product Labeling on Product Choice in Respondents with or without Celiac Disease on a GF Diet

Table 4 presents a comparison of the attributes that influenced the choice of GF bread in subgroups of celiac GF-diet and non-celiac GF-diet participants. The proportion of respondents choosing a product with an additional written disclaimer of “gluten-free” was similar for both study groups (celiac GF-diet respondents 66.2%, non-celiac GF-diet respondents 70.1%, *p* = 0.7290). Similarly, for the majority of the applied logotypes that presented additional features of the product, there was no difference between subgroups with regard to the percentage of respondents choosing a specific product. However, there was a significant difference only where a vegan logotype (“dummy” logotype not applied in Poland) was applied; the product with this logotype was chosen by 26% and 7.8% of respondents in the non-celiac GF-diet and celiac GF-diet groups, respectively (*p* = 0.0011). 

The reasons for choice of GF bread (Table 5) did not differ between the subgroups of celiac GF-diet and non-celiac GF-diet participants. A number of respondents in the non-celiac GF-diet group who selected a product with the vegan logotype did not specify vegan-friendly reasons for their choice; they either indicated other features of the product or did not specify any reason. A number of respondents specified some reasons for choosing specific products that were not only irrelevant for the purpose of this experiment, but also did not differ for the analyzed products. Based on the front-of-package labeling, participants seemingly drew conclusions about nutritional value, price, or taste, although the photographs did not present information on the nutritional value or price and the subjects did not get to taste the products.

### 3.2. Role of Age on Influence of Front-of-Package “Gluten-Free” Product Labeling on Product Choice in Respondents with or without Celiac Disease on a GF Diet

The age was revealed to be a non-significant factor for the association between front-of-package GF bread labeling and buyer choice in groups of celiac GF-diet respondents and non-celiac GF-diet respondents.

Table 6 presents a comparison of attributes that influenced choice of GF bread in subgroups of the celiac GF-diet and non-celiac GF-diet participants. The frequency of selection of specific front-of-package GF-labeled products did not differ between subgroups of celiac GF-diet participants stratified by age, as well as non-celiac GF-diet participants stratified by age.

### 3.3. Role of BMI on Influence of Front-of-Package “Gluten-Free” Product Labeling on Product Choice in Respondents with or without Celiac Disease on a GF Diet

The body mass index (BMI) was revealed to be a significant factor that modified the association between front-of-package GF bread labeling and buyer choice in group of celiac GF-diet respondents, but not for non-celiac GF-diet respondents.

Table 7 presents a comparison of attributes that influenced choice of GF bread in subgroups of the celiac GF-diet and non-celiac GF-diet participants. The frequency of selection of specific front-of-package GF-labeled products differed between subgroups of celiac GF-diet participants stratified by BMI; study participants characterized by proper body mass were less likely to select a product with no additional logotypes than other subgroups (*p* = 0.0064). The frequency of selection of specific front-of-package GF-labeled bread did not differ between subgroups of non-celiac GF-diet participants stratified by BMI.

### 3.4. Role of Place of Residence on Influence of Front-of-Package “Gluten-Free” Product Labeling on Product Choice in Respondents with or without Celiac Disease on a GF Diet

The place of residence was revealed to be a significant factor that modified the association between front-of-package GF bread labeling and buyer choice in groups of celiac GF-diet respondents and non-celiac GF-diet respondents.

Table 8 presents a comparison of attributes that influenced choice of GF bread in subgroups of the celiac GF-diet and non-celiac GF-diet participants. The frequency of selection of specific front-of-package GF-labeled products differed between subgroups of celiac GF-diet participants stratified by place of residence; study participants from cities with a population of 20,000–100,000 were more likely to select a product with the vegan logotype than other subgroups (*p* = 0.0145). The frequency of selection of specific front-of-package GF-labeled bread differed between subgroups of non-celiac GF-diet participants stratified by place of residence; study participants from villages and small towns were more likely to select a product with the European Union (EU) organic logotype than other respondents (*p* = 0.0044).

### 3.5. Role of Economic Status on Influence of Front-of-Package “Gluten-Free” Product Labeling on Product Choice in Respondents with or without Celiac Disease on a GF Diet

The economic status was revealed to be a non-significant factor for the association between front-of-package GF bread labeling and buyer choice in groups of celiac GF-diet respondents and non-celiac GF-diet respondents.

Table 9 presents a comparison of attributes that influenced choice of GF bread in subgroups of the celiac GF-diet and non-celiac GF-diet participants. The frequency of selection of specific front-of-package GF-labeled products did not differ between subgroups of celiac GF-diet participants stratified by economic status, as well as non-celiac GF-diet participants stratified by economic status.

### 3.6. Factors Influencing Front-of-Package “Gluten-Free” Product Labeling Choice in Respondents with or without Celiac Disease on a GF Diet

The additional multivariate logistic regression analysis that was conducted for all the variables (age, BMI, place of residence, economic status) revealed no combined effect of the model. The analysis was conducted for the influence on the choice of additional “gluten-free” written information—for celiac GF-diet (*p* = 0.7979; *R* = 0.2509 for a model; *p* > 0.05 for all elements of a model) and non-celiac GF-diet participants (*p* = 0.3418; *R* = 0.3453 for a model; *p* > 0.05 for all elements of a model). The separate analysis was conducted for the influence on the choice of logotypes presenting additional features—for celiac GF-diet (*p* = 0.7722; *R* = 0.2574 for a model; *p* > 0.05 for all elements of a model) and non-celiac GF-diet participants (*p* = 0.1968; *R* = 0.3804 for a model; *p* > 0.05 for all elements of a model).

Table 10 presents the summary of the obtained results of the front-of-package “gluten-free” (GF) product labeled attributes influencing the choice of GF bread in subgroups of celiac GF-diet and non-celiac GF-diet participants.

## 4. Discussion

### 4.1. Determinants of Choice of GF Products for Respondents with or without Celiac Disease on a GF Diet

The differences between consumers with and without celiac disease in a market of GF products are not widely studied [30]. Similarly, an understanding of product labeling by consumers is not commonly analyzed, despite potential differences between countries or even consumer segments [31]. Consumers with and without celiac disease who use GF products may be indicated as specific consumer segments, because their approach to GF products may differ. For patients with celiac disease and other gluten-related diseases, it is necessary to choose GF products to avoid exacerbation of disease symptoms [32]. However, for consumers of GF products who do not have celiac disease or a diagnosis of other gluten-related diseases (e.g., non-celiac gluten sensitivity, gluten ataxia), choosing products other than the GF options may be more profitable due to the possibility of higher nutritional value, as some GF products, due to their composition and applied production technology, may be characterized by a lower nutritional value [5].

The choices of specific food products are the outcomes of individual preferences and beliefs [33]. However, the declared preferences and beliefs may be disturbed by the need of consumers to present themselves in the best possible way (so-called social desirability bias) [34]. Taking this into account, the food product choice experiment (CE) is commonly applied to specify the factors that influence market choices, including the role of food product labeling—a significant resource to provide consumers information on the features of food products [35].

### 4.2. Role of “Gluten-Free” Product Labeling for Patients with or without Celiac Disease on a GF Diet

In particular, for a specific group of consumers, such as patients with celiac disease or those who follow a GF diet, front-of-package logotypes that facilitate food product labeling are of great value because inability to understand labeling may limit the possibility of dietary adherence [36]. The information provided for a GF product must be, above all, simple and easy to understand and interpret, such as the Crossed Grain Trademark (CGT), for consumers who buy products in other countries and may not know the language [37] or those who do not understand complex nutritional information, such as a nutrition information panel [38]. 

In general, the application of front-of-package labels may improve an understanding of the product value and enable the selection of more valuable food products [39,40]. In the assessed group of respondents, understanding of a “gluten-free” logotype was similar in all subgroups, both for female respondents with or without celiac disease, because the frequency of selection of products with additional written information on the lack of gluten was comparable for all the subgroups. However, the fact that a majority of respondents chose the product with additional written information may be the result of their perception that it was a safer choice in their adherence to a GF diet, which was their declared reason for choosing a specific product. There exists the possibility of a lack of trust in Poland in the “gluten-free” logotype alone, without additional written information assuring the consumer that the product is really GF. Similar observations were reported by Cornelisse-Vermaat et al. [41], who stated that respondents with food allergies from the Netherlands prefer to obtain products with written allergen information in addition to a logotype representation.

### 4.3. Role of “Free From” Product Labeling for Patients with or without Celiac Disease on a GF Diet

Currently, food product labeling, similar to that necessary for GF products, is applied for a number of product features. Labeling that is found to currently arouse a great deal of controversy includes the “free from” labels (so-called “negative claims”), which may mislead consumers by suggesting that a product without some component is better for their health than those that contain it [42]. In general, nutritional labeling may influence food choice and cause consumers to choose healthier food options [43]. However, some consumers do not understand labeling, because they automatically interpret the labeled products as a healthier choice than a product without such labeling [44]. In our study, no particular subgroup was especially prone to such suggestion; moreover, no significant differences were observed between subgroups for the tested “free from” labeling (logotypes of “dairy-free”, “wheat starch-free”, etc.).

### 4.4. Role of Vegan Product Labeling for Patients with or without Celiac Disease on a GF Diet

At the same time, a significant difference was observed for product choices between subgroups, especially for a “vegan” logotype, as products with such front-of-package labeling were commonly selected by respondents without celiac disease, compared to those with celiac disease. The popularity of such products may be the outcome of a trend toward a vegetarian or vegan diet [45]. A similar situation was reported for the GF diet because, based on the National Health and Nutrition Examination Survey (NHANES) 2009–2012, it was stated that 85% of participants who followed a GF diet were never diagnosed with celiac disease and 99% had negative serology results for celiac disease [46]. Furthermore, it may be assumed that, in this experiment, participants without celiac disease may have followed a GF diet and some additional dietary restrictions because they perceived them to be fashionable, which may be supposed as they declared following a GF diet based on their own decision, for reasons other than celiac disease or other gluten-related disease. Given that the GF diet may not be characterized by higher nutritional value than a conventional diet for such individuals, which may be confirmed by the high content of calories from sugar indicated for GF products [47], it would be especially valuable to improve the nutritional value of GF products. It should be done in order to limit the risk of individuals adopting popular diets that are not properly balanced [48]; as such, the risk of an improperly balanced diet is especially high for respondents with no medical reasons following a GF diet. 

However, in view of the previously mentioned issue of improper understanding of the information presented in the labeling, some consumers without celiac disease may subconsciously perceive GF and vegan products as more beneficial choices; therefore, they follow these diets despite the fact that they do not have to limit gluten intake in their diet. Furthermore, it may be assumed that some respondents with celiac disease who live in medium-sized cities—but neither villages nor big cities—more commonly may have chosen vegan products because they may have perceived them to be a more health-beneficial option.

### 4.5. Role of Organic Product Labeling for Patients with or without Celiac Disease on a GF Diet

Another logotype that was analyzed in this study was the EU organic logotype, which conforms to the Regulation of the European Commission No. 889/2008 [29]. For this type of front-of-package labeling, only minor differences between subgroups were stated; however, it must be emphasized that this kind of labeling is (especially in comparison with the applied “vegan” dummy logotype) the official one and guarantees the declaration of some specific product characteristics [49]. 

Organic products are, in general, similarly perceived and accepted by consumers, regardless of their age or income level [50], and this finding was proven in the present study. However, some authors indicate that organic food products may be associated with products sold in traditional retail outlets or natural product stores [51] that, in the studied group, may have been perceived as being natural products by inhabitants of villages or small towns. Thus, it may have been the reason for their more frequent selection of products with such a logotype in this subgroup of respondents without celiac disease. 

### 4.6. Limitations of the Study

Except for the interesting novel observations, in a group that is not commonly analyzed, some limitations of the study must be indicated. Due to a number of exclusion criteria and strict pair-matching, the sample size was quite small. Moreover, choice of product is in general not easy to be analyzed, as in the conducted experiment; in fact, the hypothetical declarable choice was observed, as opposed to the real one. To assess the real one, consumers would have to be observed in their real purchase decision situation. In the conducted study, consumers did not receive real products, but only the photographs; thus, the experiment may represent rather online shopping than traditional store shopping. Each product and its cues while observed is associated with specific perceptions and emotions; however, for a real product, there are more stimuli that may generate them (e.g., shape, structure, softness, etc.), than for the two-dimensional photograph of a product. At the same time, in the conducted experiment, only the front-of-package labeling was analyzed and the influence of other information on the package (e.g., nutritional value, composition, producer, etc.) was not assessed. Moreover, while the celiac GF-diet group was quite homogeneous, it must be emphasized that, for non-celiac GF-diet group, there may have been very diverse reasons for following a GF diet—ranging from perceived medical reasons of gluten exclusion (but not confirmed as a gluten-related disease) to lifestyle reasons. As a result, it must be indicated that broader studies are necessary.

## 5. Conclusions

1. The frequency of selection of products with “gluten-free” written information did not differ between subgroups of respondents with or without celiac disease; however, it was higher than the frequency of selection of product with a front-of-package GF-labeled logotype alone, and may have been the result of a higher trust in the written information than only a logotype.

2. The frequency of selection of products with additional “vegan” front-of-package GF-label was higher for respondents without celiac disease compared to those with celiac disease, and may have resulted from their vulnerability to nutritional trends that caused them to follow vegan and GF diets.

3. The frequency of selection of products with additional front-of-package GF-labeled logotype was influenced by BMI and place of residence, although not by the age and economic status of the female participants.

## Figures and Tables

**Table 1:** Characteristics of the age and body mass index of participants stratified by study groups.

		Celiac GF-Diet (*n* = 77)	Non-Celiac GF-Diet (*n* = 77)	*p*-Value **
Age (years) ^a^	Mean ± SD	34.5 ± 9.1	35.5 ± 9.9	0.4762
	Median (range)	35.0 * (20–62)	35.0 * (18–64)	
BMI (kg/m^2^) ^b^	Mean ± SD	21.7 ± 2.7	21.0 ± 2.6	0.8623
	Median (range)	21.8 (15.1–28.7)	21.6* (16.4–31.0)	

GF—gluten-free; BMI—body mass index; ^a^ for pair-matching, the difference was not allowed to be higher than five years; ^b^ for pair-matching, the difference was not allowed to be higher than 1.5 kg/ m^2^, but only within the same category of malnutrition/proper body mass/excessive body mass; * nonparametric distribution (assessed on the basis of Shapiro–Wilk test; *p* ≤ 0.05); ** Mann–Whitney U test (for nonparametric distributions).

**Table 2:** The stratification of participants by age, BMI, place of residence, and economic status.

Characteristics	Category	Celiac GF-Diet (*n* = 77)	Non-Celiac GF-Diet (*n* = 77)	*p*-Value *
Age ^a^	Young adults (≤35 years)	45 (58.4%)	39 (50.6%)	0.4183
	Adults (>35 years)	32 (41.6%)	38 (49.4%)	
BMI ^b^	Malnutrition (BMI < 18.5 kg/m^2^)	8 (10.4%)	8 (10.4%)	1.0000
	Proper body mass (BMI: 18.5–25.0 kg/m^2^)	61 (79.2%)	61 (79.2%)	
	Excessive body mass (BMI > 25.0 kg/m^2^)	8 (10.4%)	8 (10.4%)	
Place of residence ^c^	Village	5 (6.5%)	4 (5.2%)	0.9352
	Town <20,000 residents	3 (3.9%)	4 (5.2%)	
	City 20,000–100,000 residents	16 (20.8%)	14 (18.2%)	
	City >100,000 residents	53 (68.8%)	55 (71.4%)	
Economic status ^c^	Average	33 (42.9%)	20 (26.0%)	0.0751
	Good	36 (46.8%)	49 (63.6%)	
	Very good	8 (10.4%)	8 (10.4%)	

GF—gluten-free; BMI—body mass index; ^a^ category not applied for pair-matching, but only pair-matched as a continuous variable; ^b^ for pair-matching, difference between categories was not allowed; ^c^ for pair-matching, the difference was not allowed to be higher than one category; * compared using chi-square test.

**Table 3:** The scheme of the choice experiment (CE) with the detailed characteristics of products presented in a random order during the CE.

CG	Additional “Gluten-free” Written Information	Logotypes Presenting Additional Features
CG presented	“Gluten-free” information presented	-
CG presented	-	-
CG presented	“Gluten-free” information presented	EU organic logotype ^a^
CG presented	-	EU organic logotype ^a^
CG presented	“Gluten-free” information presented	“Dairy-free“ logotype ^b^
CG presented	-	“Dairy-free“ logotype ^b^
CG presented	“Gluten-free” information presented	Wheat starch free logotype ^b^
CG presented	-	Wheat starch free logotype ^b^
CG presented	“Gluten-free” information presented	Quality logotype ^c^
CG presented	-	Quality logotype ^c^
CG presented	“Gluten-free” information presented	Vegan logotype ^c^
CG presented	-	Vegan logotype ^c^

CG—Crossed Grain as in accordance with Crossed Grain Trademark [28]; EU—European Union; ^a^ according to the Regulation of European Commission No 889/2008 [29]; ^b^ logotypes commonly applied for products available in Poland; ^c^ “dummy” logotypes not applied in Poland.

**Table 4:** The comparison of the front-of-package “gluten-free” (GF) product labeled attributes influencing the choice of GF bread in subgroups of celiac GF-diet and non-celiac GF-diet participants.

		Celiac GF-Diet (*n* = 77)	Non-Celiac GF = Diet (*n* = 77)	*p*-Value *
Additional “gluten-free” written information **	Logotype with no written information	26 (33.8%)	23 (29.9%)	0.7290
	Logotype with written information	51 (66.2%)	54 (70.1%)	
Logotypes presenting additional features **	No additional feature logotypes	8 (10.4%)	8 (10.4%)	1.0000
	EU organic logotype ^a^	21 (27.3%)	15 (19.5%)	0.3412
	“Dairy-free“ logotype ^b^	18 (23.4%)	17 (22.1%)	0.8475
	Wheat starch-free logotype ^b^	7 (9.1%)	3 (3.9%)	0.3264
	Quality logotype ^c^	17 (22.1%)	14 (18.2%)	0.6873
	Vegan logotype ^c^	6 (7.8%)	20 (26.0%)	0.0011

GF—gluten-free; EU—European Union; ^a^ according to the Regulation of European Commission No 889/2008 [29]; ^b^ logotypes commonly applied for products available in Poland; ^c^ “dummy” logotypes not applied in Poland; * compared using chi-square test; ** single choice based on indicated product.

**Table 5:** The reasons of choice of “gluten-free” (GF) bread in subgroups of celiac GF-diet and non-celiac GF-diet participants (multiple choice based on open-ended question).

		Celiac GF-Diet (*n* = 77)	Non-Celiac GF-Diet (*n* = 77)	*p*-Value *
Associated with gluten-free diet	Gluten-free diet	29 (37.7%)	29 (37.7%)	1.0000
	Safe choice	6 (7.8%)	5 (6.5%)	1.0000
Associated with presented logotypes	Eco-conscious product	8 (10.4%)	8 (10.4%)	1.0000
	Lactose-free diet	12 (15.6%)	12 (15.6%)	1.0000
	Wheat-free product	2 (2.6%)	1 (1.3%)	1.0000
	High-quality product	12 (15.6%)	9 (11.7%)	0.6390
	Vege-friendly product	4 (5.2%)	5 (6.5%)	1.0000
Associated with features articulated by respondents	Packaging design	8 (10.4%)	6 (7.8%)	0.7794
	Other reasons	5 (6.5%)	6 (7.8%)	1.0000
Not declared		20 (26.0%)	21 (27.3%)	0.8568

GF—gluten-free; * compared using chi-square test.

**Table 6:** The comparison of the front-of-package “gluten-free” (GF) product labeled attributes influencing the choice of GF bread in subgroups of celiac GF-diet and non-celiac GF-diet participants stratified by age.

Characteristics		Young Adults (≤ 35 years)	Adults (> 35 years)	*p*-Value *
**Celiac GF-diet**				
Additional “gluten-free“ written information **	Logotype with no written information	35.6%	31.3%	0.8820
	Logotype with written information	64.4%	68.7%	
Logotypes presenting additional features **	No additional feature logotypes	11.1%	9.4%	0.8932
	EU organic logotype ^a^	24.4%	31.2%	0.6882
	“Dairy-free“ logotype ^b^	28.9%	15.6%	0.2792
	Wheat starch-free logotype ^b^	8.9%	9.4%	0.7424
	Quality logotype ^c^	22.3%	21.9%	0.8081
	Vegan logotype ^c^	4.4%	12.5%	0.3852
**Non-celiac GF-diet**				
Additional “gluten-free“ written information **	Logotype with no written information	35.9%	23.7%	0.3568
	Logotype with written information	64.1%	76.3%	
Logotypes presenting additional features **	No additional feature logotypes	7.7%	13.2%	0.7379
	EU organic logotype ^a^	20.5%	18.4%	0.9563
	“Dairy-free“ logotype ^b^	20.5%	23.7%	0.9496
	Wheat starch-free logotype ^b^	0.0%	7.8%	0.2481
	Quality logotype ^c^	23.1%	13.2%	0.3471
	Vegan logotype ^c^	28.2%	23.7%	0.7436

GF—gluten-free; EU—European Union; ^a^ according to the Regulation of European Commission No 889/2008 [29]; ^b^ logotypes commonly applied for products available in Poland; ^c^ “dummy” logotypes not applied in Poland; * compared using chi-square test; ** single choice based on indicated product.

**Table 7:** The comparison of the front-of-package “gluten-free” (GF) product labeled attributes influencing the choice of GF bread in subgroups of celiac GF-diet and non-celiac GF-diet participants stratified by BMI.

Characteristics		Malnutrition (BMI <18.5 kg/m^2^)	Proper Body Mass (BMI 18.5–25.0 kg/m^2^)	Excessive Body Mass (BMI >25.0 kg/m^2^)	*p*-Value *
**Celiac GF-diet**					
Additional “gluten-free“ written information **	Logotype with no written information	37.5%	29.5%	62.5%	0.1740
	Logotype with written information	62.5%	70.5%	37.5%	
Logotypes presenting additional features **	No additional feature logotypes	37.5%	4.9%	25.0%	0.0064
	EU organic logotype ^a^	25.0%	29.5%	12.5%	0.7265
	“Dairy-free“ logotype ^b^	25.0%	26.2%	0.0%	0.2554
	Wheat starch-free logotype ^b^	12.5%	6.6%	25.0%	0.2191
	Quality logotype ^c^	0.0%	24.6%	25.0%	0.2822
	Vegan logotype ^c^	0.0%	8.2%	12.5%	0.6260
**Non-celiac GF-diet**					
Additional “gluten-free“ written information **	Logotype with no written information	12.5%	34.4%	12.5%	0.2335
	Logotype with written information	87.5%	65.6%	87.5%	
Logotypes presenting additional features **	No additional feature logotypes	0.0%	11.5%	12.5%	0.5975
	EU organic logotype ^a^	12.5%	19.6%	25.0%	0.8167
	“Dairy-free“ logotype ^b^	37.5%	21.3%	12.5%	0.4600
	Wheat starch-free logotype ^b^	0.0%	3.3%	12.5%	0.3738
	Quality logotype ^c^	37.5%	14.8%	25.0%	0.2542
	Vegan logotype ^c^	12.5%	29.5%	12.5%	0.3854

GF—gluten-free; BMI—body mass index; EU—European Union; ^a^ according to the Regulation of European Commission No 889/2008 [29]; ^b^ logotypes commonly applied for products available in Poland; ^c^ “dummy” logotypes not applied in Poland; * compared using chi-square test; ** single choice based on indicated product.

**Table 8:** The comparison of the front-of-package “gluten-free” (GF) product labeled attributes influencing the choice of GF bread in subgroups of celiac GF-diet and non-celiac GF-diet diet participants stratified by place of residence.

Characteristics		Village or Town <20,000 Residents	City 20,000–100,000 Residents	City >100,000 Residents	*p*-Value *
**Celiac GF-diet**					
Additional “gluten-free“ written information **	Logotype with no written information	37.5%	31.2%	34.0%	0.9905
	Logotype with written information	62.5%	68.8%	66.0%	
Logotypes presenting additional features **	No additional feature logotypes	12.5%	12.5%	9.4%	0.9199
	EU organic logotype ^a^	12.5%	18.7%	32.2%	0.3531
	“Dairy-free“ logotype ^b^	37.5%	12.5%	24.5%	0.3703
	Wheat starch-free logotype ^b^	12.5%	12.5%	7.5%	0.7827
	Quality logotype ^c^	25.0%	18.7%	22.6%	0.8976
	Vegan logotype ^c^	0.0%	25.1%	3.8%	0.0145
**Non-celiac GF-diet**					
Additional “gluten-free“ written information **	Logotype with no written information	25.0%	21.4%	32.7%	0.6767
	Logotype with written information	75.0%	78.6%	67.3%	
Logotypes presenting additional features **	No additional feature logotypes	12.5%	14.3%	9.1%	0.8156
	EU organic logotype ^a^	50.0%	7.1%	18.2%	0.0044
	“Dairy-free“ logotype ^b^	0.0%	14.3%	27.3%	0.1633
	Wheat starch-free logotype ^b^	0.0%	7.1%	3.6%	0.6949
	Quality logotype ^c^	12.5%	28.6%	16.4%	0.5189
	Vegan logotype ^c^	25.0%	28.6%	25.4%	0.9700

GF—gluten-free; EU—European Union; ^a^ according to the Regulation of European Commission No 889/2008 [29]; ^b^ logotypes commonly applied for products available in Poland; ^c^ “dummy” logotypes not applied in Poland; * compared using chi-square test; ** single choice based on indicated product.

**Table 9:** The comparison of the front-of-package “gluten-free” (GF) product labeled attributes influencing the choice of GF bread in subgroups of celiac GF-diet and non-celiac GF-diet participants stratified by economic status.

Characteristics		Average	Good or Very Good	*p*-Value *
**Celiac GF-diet**				
Additional “gluten-free“ written information **	Logotype with no written information	36.4%	31.8%	0.8645
	Logotype with written information	63.6%	68.2%	
Logotypes presenting additional features **	No additional feature logotypes	12.1%	9.1%	0.9563
	EU organic logotype ^a^	33.3%	22.7%	0.4378
	“Dairy-free“ logotype ^b^	18.2%	27.3%	0.5086
	Wheat starch-free logotype ^b^	9.1%	9.1%	0.6892
	Quality logotype ^c^	21.2%	22.7%	0.8744
	Vegan logotype ^c^	6.1%	9.1%	0.9203
**Non-celiac GF-diet**				
Additional “gluten-free“ written information **	Logotype with no written information	40.0%	26.3%	0.3862
	Logotype with written information	60.0%	73.7%	
Logotypes presenting additional features **	No additional feature logotypes	10.0%	10.5%	0.7195
	EU organic logotype ^a^	25.0%	17.5%	0.6919
	“Dairy-free“ logotype ^b^	30.0%	19.4%	0.5967
	Wheat starch-free logotype ^b^	5.0%	3.5%	0.7072
	Quality logotype ^c^	20.0%	17.5%	0.9287
	Vegan logotype ^c^	10.0%	31.6%	0.0583

GF—gluten-free; EU—European Union; ^a^ according to the Regulation of European Commission No 889/2008 [29]; ^b^ logotypes commonly applied for products available in Poland; ^c^ “dummy” logotypes not applied in Poland; * compared using chi-square test; ** single choice based on indicated product.

**Table 10:** The summary of the obtained results of the front-of-package “gluten-free” (GF) product labeled attributes influencing the choice of GF bread in subgroups of celiac GF-diet and non-celiac GF-diet participants.

	Celiac GF-diet			Non-celiac GF-diet		
**Age**	Lack of influence			Lack of influence		
**BMI**	Malnutrition	Proper body mass	Excessive body mass	Lack of influence		
	↓ no additional logotypes	↑ no additional logotypes	↓ no additional logotypes			
**Place of residence**	Villages and small towns	Medium-sized towns	Big cities	Villages and small towns	Medium-sized towns	Big cities
	↓ vegan logotype	↑ vegan logotype	↓ vegan logotype	↑ organic logotype	↓ organic logotype	↓ organic logotype
**Economic status**	Lack of influence			Lack of influence		

↓—chosen less often; ↑—chosen more often.
